# Supplementary material for: Age-dependent changes in the anatomical and histological characteristics of the aggregated lymphoid nodules in the stomach of Dromedary camels (Camelus Dromedarius)
Source: PLoS One. 2023 Mar 22;18(3):e0279417. doi: 10.1371/journal.pone.0279417 (PMC10032504; doi:10.1371/journal.pone.0279417)
Supplement: Attachment — Submitted filename: Response to Reviewers.docx [file pone.0279417.s001.docx]

Response to Reviewers' comments:

Comment.1

Has the statistical analysis been performed appropriately and rigorously?

Reviewer #1: No

Reviewer #2: Yes

**Response:** Thank you for this suggestion, we appreciate your comment. We have changed the way we analyzed the data please see figure 5 and 6.

Comment.2

Is the manuscript presented in an intelligible fashion and written in standard English?
Reviewer #1: No

Reviewer #2: No

**Response:** We are grateful for your comment. We have revised the language and corrected all grammatical and typing error. The manuscript has been revised by a native English speaker.

Comment.3

The apt title of the study according to the presented results shown in the manuscript would be ‘Age-dependent changes in the anatomical and histological characteristics of the aggregated lymphoid nodules in the stomach of Dromedary camels. The study of age-dependent changes of ALNA are very interesting.

**Response**: Agreed. Thank you for this valuable comment, we have changed the title “*Age-dependent changes in the anatomical and histological characteristics of the aggregated lymphoid nodules in the stomach of Dromedary camels (Camelus Dromedarius)****”***

Comment.4

Although the manuscript is readable, understandable, and scientifically sound, the authors are suggested to carefully check and revise grammatical and typing errors in the whole manuscript. For example, in line 30, it is written that Gastrointestinal associated lymphoid tissue (GALT) is important component of the mucosal immune system, while an article is missing in the sentence. It should be written as Gastrointestinal associated lymphoid tissue (GALT) is an important component of the mucosal immune system. In another example, in lines 50-52, it is written as ‘The histological characteristics of the structure was the same as PPs in the intestine of dromedary camel while its anatomical appearance showed some dissimilarity.’ It should be written like ‘The histological characteristics of the structure were the same as PPs in the intestine of the dromedary camel, while anatomical appearance showed some difference.’ The use of articles and commas is missing. In line 229, the full stop is missing. Please also check extra spaces and carefully revise the manuscript.

**Response**: thank you very much for these comments. we have carefully checked and corrected this part please see line 28 and line (48~50). We also checked extra spaces.

Comment.5

Why the authors have used unnecessary hyphen (-), e.g., in line 92: nod-ules, line 155: de-creasing, in line 159: Ja-pan, and at other places there are many more such types of hyphens used? Any reason?

**Response**: Thank you very much for these important notices. We have corrected these mistakes.

Comment.6

In line 153, what is the fixed volume size of tissue block in mm3? Any number?

**Response**: Thank you, we have added the volume size of tissue block (1mm^3^). Please see line (150).

Comment.7

In fig. 2, please show a triangular zone either with the dotted line triangular zone or an arrow as according to the written sentences in lines 175-176.

**Response**: Thank you, we have shown the triangular zone with arrows. please see figure 2.

Comment.8

In fig. 1-4, the calibration bar is missing. Please increase the font size of the calibration label in fig. 7-9.

**Response**: Agreed. We have increased the font size of the calibration label in fig. 7-9.

Comment.9

In fig. 9 legends, magnification of microscope objective is written 400x. Is it true?

**Response**: the magnification of the microscopic objective is 400x, we observed it in the microscope using the lens 40.

Comment.10

In line, 258, what does mean by HIV. Please clarify the abbreviation so that non-expert can understand.

**Response**: Thank you for this important comment. We have corrected the mistake. Please see line 255

Comment.11

In line 275, the term ‘department’ should be replaced with ‘compartment’.

**Response:** Agreed. Please See line 273.

Comment.12

In statistical analysis, fig. 5 and fig. 6, the authors did not show the significant relationships clearly. The authors are suggested to use a line bar along with streak(s) (*) at the top of graph to show significant relationships between experimental subjects.

**Response**: thank you very much for much for this valuable comments. We have changed the way we analyzed the data. Please see figure 5 and 6.

Comment.13

In the introduction, the authors mentioned that such lymphoid tissues had been previously identified in the abomasum of Bactrian camel apart from pigs. In my opinion, the same observation in another species makes the discovery redundant.

**Response**: Agreed. We have removed this part from the introduction.

Comment.14

To confirm presence of the lymphoid nodules, the experiments undertaken by the authors are solely anatomical and histological in nature. However, no molecular characterization was carried out in order to confirm the lymphoid status, for example, presence of lymphocytes, etc. This makes me wonder whether this manuscript falls under the purview of Plos One, or is more appropriate for a journal publishing anatomical/histological characterizations.

**Response**: We are grateful for your suggestion. we have done the immunohistochemistry and detected the immunoglobulin–producing cells please see the results (section 3.5).
