# Supplementary material for: Age-dependent changes in the anatomical and histological characteristics of the aggregated lymphoid nodules in the stomach of Dromedary camels (Camelus Dromedarius)
Source: PLoS One. 2023 Mar 22;18(3):e0279417. doi: 10.1371/journal.pone.0279417 (PMC10032504; doi:10.1371/journal.pone.0279417)
Supplement: Attachment — Submitted filename: Response to Reviewers..docx [file pone.0279417.s002.docx]

Response to Reviewers' comments:

Comment.1

Has the statistical analysis been performed appropriately and rigorously?

Reviewer #1: No

Reviewer #2: Yes

**Response:** Thank you, We have addressed Reviewer 1 comment, please see the statistical results and figures.

Comment.2

Is the manuscript presented in an intelligible fashion and written in standard English?
Reviewer #1: No

Reviewer #2: Yes

**Response:** We are grateful for your comment. We have revised the language. The manuscript has been revised by a native English speaker.

Review Comments to the Author

Reviewer #1: The revised manuscript is improved; however, its presentation quality still does not match the standard of the journal. Although scientific data are organized and conclusions are in the line of results, without rigorous and clear statistical analysis, results are difficult to conclude. Additionally, the representation of results is not consistent. Therefore, the authors are strongly suggested to revise the manuscript very carefully before its final decision to be accepted for publication.

Major issue:

1. The authors have mentioned that the statistically significant differences in the width and height of the reticular and longitudinal mucosal folds of the abomasum ALNA between groups; were determined by one-way analysis of variance (ANOVA). However, statistical significance is not clearly described either in the text or in the figure legends. Based on p-values, no streak (*) can be seen in any figure.

**Response**: Agreed. Thank you for this valuable comment, we have described the statistical significance in both the text and in the figure’s legends. Streaks have been shown. Please see the figures.

2. In lines 331– 333, it is mentioned- ‘However, there were significant differences………. but there was no significant difference between young and pubertal groups (P < 0.0001).’ This indicates either the p-value is written at the wrong place or the interpretation of data is wrong.

**Response**: Thank you for drawing our attention to this . This part has been addressed accordingly.

Minor issues:

1. Fig. 1 – 4 have no calibration bar.

Response: Thank you very much for this comment. We would like to inform that these figures has been taken from the field by normal camera.

2. In line, the volume of a block should be written as 1 cm^3^ instead of 1 cm. Please write an appropriate unit.

**Response**: please the correction has been made in the manuscript at line 144.

3. Fig. 7 and 8 – the calibration bar and its labeling are not consistent for all three figures.

**Response**: Thank you for this comment.The figure title has been corrected. The calibration bars were correct, all these figures were taken using the lens 4. please See line (272 – 276) and (282 – 285).

4. In fig. 5 and 6, what is the meaning of alphabets- a, b, c…….. Please use streak to represent significance.

**Response**: Bars with a different letter are significantly different. Streaks has been shown please see the figures.

5. Fig. 10 and 11 – Difficult to see calibration bar. Please describe red rectangles in the figure legend.

**Response**: Thank you. This time our figures are clear. Red rectangle has been described in the figure legend.

Reviewer #2: To address the concerns of this reviewer, the authors have performed immunohistochemistry experiments to confirm the lymphoid status of the nodules. I have no further concerns and recommend this manuscript for publication.

**Response**: We are grateful to you . Thanks to the all reveiwers for their time and comments. We hope our manuscript would finally meet all reviewer’s favorable consideration and approval to be published in this highly respected journal.
